# Metformin Cessation and Dementia Incidence

**DOI:** 10.1001/jamanetworkopen.2023.39723

**Published:** 2023-10-25

**Authors:** Scott C. Zimmerman, Erin L. Ferguson, Vidhu Choudhary, Dilrini K. Ranatunga, Akinyemi Oni-Orisan, Eleanor Hayes-Larson, Aline Duarte Folle, Elizabeth Rose Mayeda, Rachel A. Whitmer, Paola Gilsanz, Melinda C. Power, Catherine Schaefer, M. Maria Glymour, Sarah F. Ackley

**Affiliations:** 1 Department of Epidemiology and Statistics, University of California, San Francisco; 2 Kaiser Permanente Division of Research, Oakland, California; 3 Now with Kaiser Permanente Research Bank, Oakland, CA; 4 Department of Clinical Pharmacy, University of California, San Francisco; 5 Department of Epidemiology, Fielding School of Public Health, University of California, Los Angeles; 6 Department of Public Health Sciences, University of California, Davis; 7 Department of Epidemiology, George Washington University Milken Institute School of Public Health, Washington, District of Columbia; 8 Department of Epidemiology, Boston University, Boston, Massachusetts

## Abstract

**Importance:**

Prior studies suggested that metformin may be associated with reduced dementia incidence, but associations may be confounded by disease severity and prescribing trends. Cessation of metformin therapy in people with diabetes typically occurs due to signs of kidney dysfunction but sometimes is due to less serious adverse effects associated with metformin.

**Objective:**

To investigate the association of terminating metformin treatment for reasons unrelated to kidney dysfunction with dementia incidence.

**Design, Setting, and Participants:**

This cohort study was conducted at Kaiser Permanente Northern California, a large integrated health care delivery system, among a cohort of metformin users born prior to 1955 without history of diagnosed kidney disease at metformin initiation. Dementia follow-up began with the implementation of electronic health records in 1996 and continued to 2020. Data were analyzed from November 2021 through September 2023.

**Exposures:**

A total of 12 220 early terminators, individuals who stopped metformin with normal estimated glomerular filtration rate (eGFR), were compared with routine metformin users, who had not yet terminated metformin treatment or had terminated (with or without restarting) after their first abnormal eGFR measurement. Early terminators were matched with routine users of the same age and gender who had diabetes for the same duration.

**Main outcomes and measures:**

The outcome of interest was all-cause incident dementia. Follow-up for early terminators and their matched routine users was started at age of termination for the early terminator. Survival models adjusted for sociodemographic characteristics and comorbidities at the time of metformin termination (or matched age). Mediation models with HbA_1c_ level and insulin usage 1 and 5 years after termination tested whether changes in blood glucose or insulin usage explained associations between early termination of metformin and dementia incidence.

**Results:**

The final analytic sample consisted of 12 220 early terminators (5640 women [46.2%]; mean [SD] age at start of first metformin prescription, 59.4 [9.0] years) and 29 126 routine users (13 582 women [46.6%]; mean [SD] age at start of first metformin prescription, 61.1 [8.9] years). Early terminators had 1.21 times the hazard of dementia diagnosis compared with routine users (hazard ratio, 1.21; 95% CI, 1.12 to 1.30). In mediation analysis, contributions to this association by changes in HbA_1c_ level or insulin use ranged from no contribution (0.00 years; 95% CI, −0.02 to 0.02 years) for insulin use at 5 years after termination to 0.07 years (95% CI, 0.02 to 0.13 years) for HbA_1c_ level at 1 year after termination, suggesting that the association was largely independent of changes in HbA_1c_ level and insulin usage.

**Conclusions and Relevance:**

In this study, terminating metformin treatment was associated with increased dementia incidence. This finding may have important implications for clinical treatment of adults with diabetes and provides additional evidence that metformin is associated with reduced dementia risk.

## Key Points

**Question:**

Is cessation of metformin therapy associated with dementia incidence, and is the association mediated by hemoglobin A_1c_ (HbA_1c_) level or insulin use?

**Findings:**

This cohort study of 12 220 early terminators and 29 126 routine users of metformin found that cessation of metformin therapy without abnormal kidney function markers was associated with 1.21 times the hazard of dementia diagnosis compared with continuation of therapy or cessation with abnormal kidney function markers. This association was minimally mediated by increases in HbA_1c_ level and not mediated by insulin use 1 or 5 years after metformin cessation.

**Meaning:**

The findings of this study suggest that metformin cessation is associated with increased dementia incidence and that mechanisms other than glucose control or insulin use may mediate this association.

## Teaser

This cohort study investigates the association of metformin therapy cessation with dementia incidence and mediation of this association by hemoglobin A_1c_ level and insulin use.

## Introduction

Type 2 diabetes occurs among an increasing fraction of people aged older than 65 years in the US, and diabetes is associated with increased dementia risk.^1,2^ Approved in 1995 in the US,^3^ metformin (dimethylbiguanide) has been the preferred first-line agent for type 2 diabetes since 2006.^4,5,6^ Metformin treatment reduces incidence of diabetes complications and diabetes-related and all-cause mortality.^7^ Metformin may also reduce dementia risk by improved glucose control or by mechanisms unrelated to diabetes, including activation of adenosine monophosphate–activated protein kinase, which may mimic starvation,^7^ or by inhibition of aromatase, which may be associated with lower blood pressure.^8,9^

Previous randomized clinical trials found that metformin treatment improved cognition and lowered dementia risk in people with type 2 diabetes, but this may reflect cognitive benefits of glucose lowering independent of the agent used.^10^ In contrast, the Action to Control Cardiovascular Risk in Diabetes-Memory in Diabetes (ACCORD-MIND) randomized clinical trial^11,12^ found no evidence of cognitive benefit for an intensive glucose-control strategy (glycated hemoglobin A_1c_ [HbA_1c_] target level: <6.0%), which increased exposure to antidiabetes drugs, including metformin, compared with an HbA_1c_ target level of 7.0% to 7.9%. Prior observational studies found that initiating metformin treatment was associated with a benefit in dementia risk, including a benefit compared with other antidiabetes drugs.^10,13^ However, confounding by diabetes severity and duration may bias associations.^10^ Additionally, increasing metformin use^14,15^ and decreasing age-specific dementia incidence in the US^16^ complicate observational comparisons between metformin and other agents.^10^ Furthermore, some prior studies compared prevalent users with nonusers, which can lead to immortal person-time and confounding biases.^17^

Individuals may terminate metformin for several reasons.^18,19^ Gastrointestinal adverse effects are more common with metformin than with other antidiabetes agents,^20^ leading to lower adherence^18^ and replacement with other antidiabetes agents in one-fifth of early users.^21,22^ Metformin may also be terminated because it is associated with increased mortality in people with kidney dysfunction, a common type 2 diabetes complication.^21,23,24^ Current recommendations are to discontinue metformin when the estimated glomerular filtration rate (eGFR) decreases to less than 30 or 45 mL/min/1.73 m^2^ of height, depending on benefits and risks of continued use.^3^ Examining metformin cessation may mitigate confounding by disease severity and cohort effects associated with studies of metformin initiation.

We evaluated the association between termination of metformin treatment for reasons unrelated to kidney dysfunction and dementia incidence using an emulated trial design.^25,26^ We compared individuals in the Kaiser Permanente Northern California (KPNC) health care system who terminated metformin without abnormal kidney function markers with individuals who continued metformin therapy or stopped after abnormal kidney function. We investigated whether metformin was associated with reduced risk of dementia and whether this association was mediated predominantly by mechanisms other than improved glucose control or insulin use.

## Methods

This cohort study followed the Strengthening the Reporting of Observational Studies in Epidemiology (STROBE) reporting guideline.^33^ Human participant approval was granted by University of California, San Francisco, and KPNC, Mid-Atlantic States institutional review boards. All participants provided informed consent.

### Setting and Data

KPNC is an integrated health care delivery system with 4.6 million members.^27^ Older adult KPNC members are similar to the general older adult population of Northern California with respect to chronic conditions (diabetes, hypertension, heart disease, and asthma), other risk factors (smoking, obesity, and sedentary behavior),^28^ and demographics; extremes of the income distribution are underrepresented.^28,29,30^

This study used deidentified survey results and electronic health records from KPNC members who had used metformin; were dementia free; were born prior to January 1, 1955; and had completed 1 of 2 harmonized health surveys: (1) the California Men’s Health Survey,^31^ offered 2002 to 2003 to men aged 45 to 69 years in January 2000 who had been KPNC members for at least 1 year, and (2) the Kaiser Permanente Research Program on Genes, Environment, and Health survey, offered 2007 to 2009 to adults who had been KPNC members for at least 2 years.^32^ Dementia follow-up began with the implementation of electronic health records in 1996 and continued to 2020.

### Follow-Up

Participants were followed up from the first availability of electronic health records (starting in 1996) until administrative censoring, age 90 years, death, the start of a 90-day membership gap, or dementia diagnosis. Each participant was assigned an administrative censoring date randomly chosen between January 30, 2020, and March 31, 2020, so that date of birth could not be inferred. Death dates were obtained from the KPNC mortality database, which combines KPNC clinical and administrative, National Death Index, California State death, and Social Security Administration records. Details on diagnostic, laboratory, prescription record, diabetes registry, and sociodemographic variables are given in the eMethods in Supplement 1 and eTables 8 and 9 Supplement 2. Race and ethnicity were self-reported. See original race and ethnicity categories, along with other details on race and ethnicity reporting, in the eMethods in Supplement 1. These were collapsed into the following categories for consistency between survey and other data sources and because participants could endorse multiple categories: Asian, Black, Hispanic or Latino, White, and other or uncertain. An algorithm (validated by comparing against several sources, including the KPNC virtual data warehouse) was used to assign 1 adjudicated race or ethnicity category to individuals who endorsed more than 1 category, with the following prioritization order: Black, Hispanic, Asian, and Native American. Race and ethnicity was derived from KPNC health plan databases for individuals who endorsed no categories. Race and ethnicity were assessed by KPNC to improve patient care and for research purposes.

### Metformin Cessation Groups

Exposures were categorized as follows: First, the age of first documented abnormal kidney function after initiating metformin was determined for each individual in a cohort of metformin users. Early terminators were defined as individuals who stopped using metformin without prior history of abnormal kidney function. A routine user was any individual who remained on metformin at the age when the matched early terminator ceased using metformin. No information from times after the cessation time was used to define exposure status. Follow-up for routine users ended at censoring or when the individual became an early terminator. We excluded individuals who stopped using metformin but had no antidiabetes medication prescriptions within 1 month, given that prescription nonadherence may be a sign of incipient dementia or particularly good control of blood glucose with nonpharmacologic interventions. Such individuals would not be comparable to individuals who remained on metformin.

### Statistical Analysis

**Figure 1.**
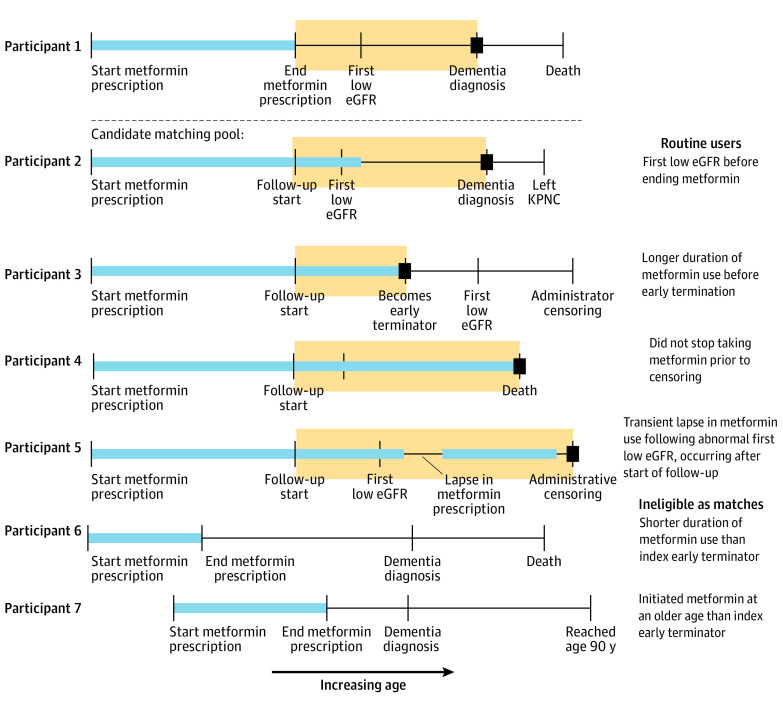
Matching Procedure Showing Timelines for 7 Hypothetical Study Participants The timeline for participant 1 represents the index, the early terminator for whom matches were sought. Other timelines represent the candidate pool of individuals matched exactly to the index based on age at metformin initiation, gender, and whether metformin was the first diabetes drug prescribed in the Kaiser Permanente Northern California (KPNC) health care system. Orange boxes indicate the person-time that would be contributed for each participant if matched, with follow-up time starting at the age of metformin termination for the index. Blue boxes indicate metformin prescriptions, and black squares indicate the end of contributed person-time due to censoring. eGFR indicates estimated glomerular filtration rate.

Analyses were conducted using R statistical software version 4.0.4 (R Project for Statistical Computing). All *P* values calculated were 2-sided, with a significance threshold of α = .05. Data were analyzed from November 2021 through September 2023. Figure 1 and eFigure 1 in Supplement 1 provide a conceptual model of the emulated trial design used. An emulated trial design is intended to replicate key features of a randomized trial. Estimated associations are analogous to intent-to-treat estimates given that we compared early terminators with the routine user group, which includes individuals who stopped taking metformin after kidney dysfunction markers were found. Each early terminator was matched with up to 4 individuals using incidence density sampling. For the purposes of the match, these individuals were routine users, but they could go on to be early terminators themselves at an older age. Matching was performed such that no matches were repeated, with exact matching on gender and whether metformin was the first diabetes drug prescribed in KPNC. A distance measure was calculated with age of metformin initiation, diabetes duration (years since first diagnosis of diabetes in the registry), and HbA_1c_ level using the sum of absolute differences, with HbA_1c_ level multiplied by 10 to be on approximately the same scale as age. Among individuals eligible based on exact matching criteria, matches were assigned by selecting a routine user with the minimum distance to the early terminator. Matches were rejected if matched pairs ages of metformin initiation differed by more than 5 years, the difference in HbA_1c_ level was greater than 0.5%, or the difference in diabetes duration was greater than 5 years. If no match meeting these criteria could be obtained for an early terminator, that individual was excluded from the analysis. Matches were iteratively chosen such that early terminators received matches before being considered for subsequent matches. To prevent immortal person-time bias, an early terminator and the matched routine user began follow-up time at the age that the early terminator stopped metformin treatment. Individuals were not eligible for matching after dementia diagnosis.

We used Cox proportional hazards models to estimate hazard ratios (HRs) and 95% CIs of dementia diagnosis using time since metformin termination (or equivalent for routine users) as the time scale. To provide a simple interpretation of associations, we also present HRs and 95% CIs from accelerated failure time (AFT) models with a Weibull-distributed waiting time. We present HRs adjusted only for matching variables (minimally adjusted models) and HRs additionally adjusted for demographic characteristics and comorbidities at the time of metformin initiation (fully adjusted models).

#### Covariates

For matched pairs, covariates were ascertained at the age of the early terminator’s metformin cessation. Models adjusted for demographic characteristics (gender, age at metformin initiation, race and ethnicity, educational attainment, nativity, parental nativity, and survey language) and low-density lipoprotein and HbA_1c_ levels at the time of metformin termination. We additionally adjusted for history of abnormal kidney function prior to metformin initiation as a binary variable, baseline use of antilipemic agents, insulin use, nonmetformin oral hypoglycemic use, diabetes status, and whether metformin was the first diabetes drug prescribed. We adjusted for cardiovascular disease history and number of categories of events (hypertension or secondary hypertension, ischemic heart disease, heart attack, and stroke) and cancer history .

#### Causal Mediation Analysis

Causal mediation analysis^34,35,36^ assessed whether the association between early metformin termination and dementia was mediated by changes in HbA_1c_ level or insulin prescription status measured approximately 1 year after early metformin termination (specifically, mean HbA_1c_ level during the period 8-16 months after termination) or at the same age among matched routine users. Individuals who got dementia, died, were censored prior to mediator measurement, or did not have mediators measured were excluded from mediation analyses. In additional analyses, we used HbA_1c_ level (specifically, mean levels at 4 years, 8 months to 5 years, 4 months) and change in insulin prescription status at 5 years after early metformin termination as mediators. Mediation analyses used AFT models with linear mediator models with the same covariates as the fully adjusted model. We assessed for exposure-mediator interaction in outcome models with a prespecified threshold of α = .05 for significance. We calculated total, natural direct, and natural indirect effects of metformin termination on time to dementia and 95% CIs.

The following sensitivity analyses were performed: First, we repeated analyses using creatinine instead of eGFR. Gender-specific cutoffs of 1.4 mg/dL (124 μmol/L) or greater for women and 1.5 mg/dL (133 μmol/L) or greater for men were used. Second, we restricted analysis to participants with medication adherence exceeding 80% (eMethods in Supplement 1). Third, we further restricted differences between early and routine users, rejecting matches with differences greater than 0.1% in HbA_1c_ level or 1 year of diabetes duration. Finally, we restricted analysis to early terminators who had initiated metformin within the prior 2 years, a time frame too short for diabetes progression to be likely.

## Results

**Figure 2.**
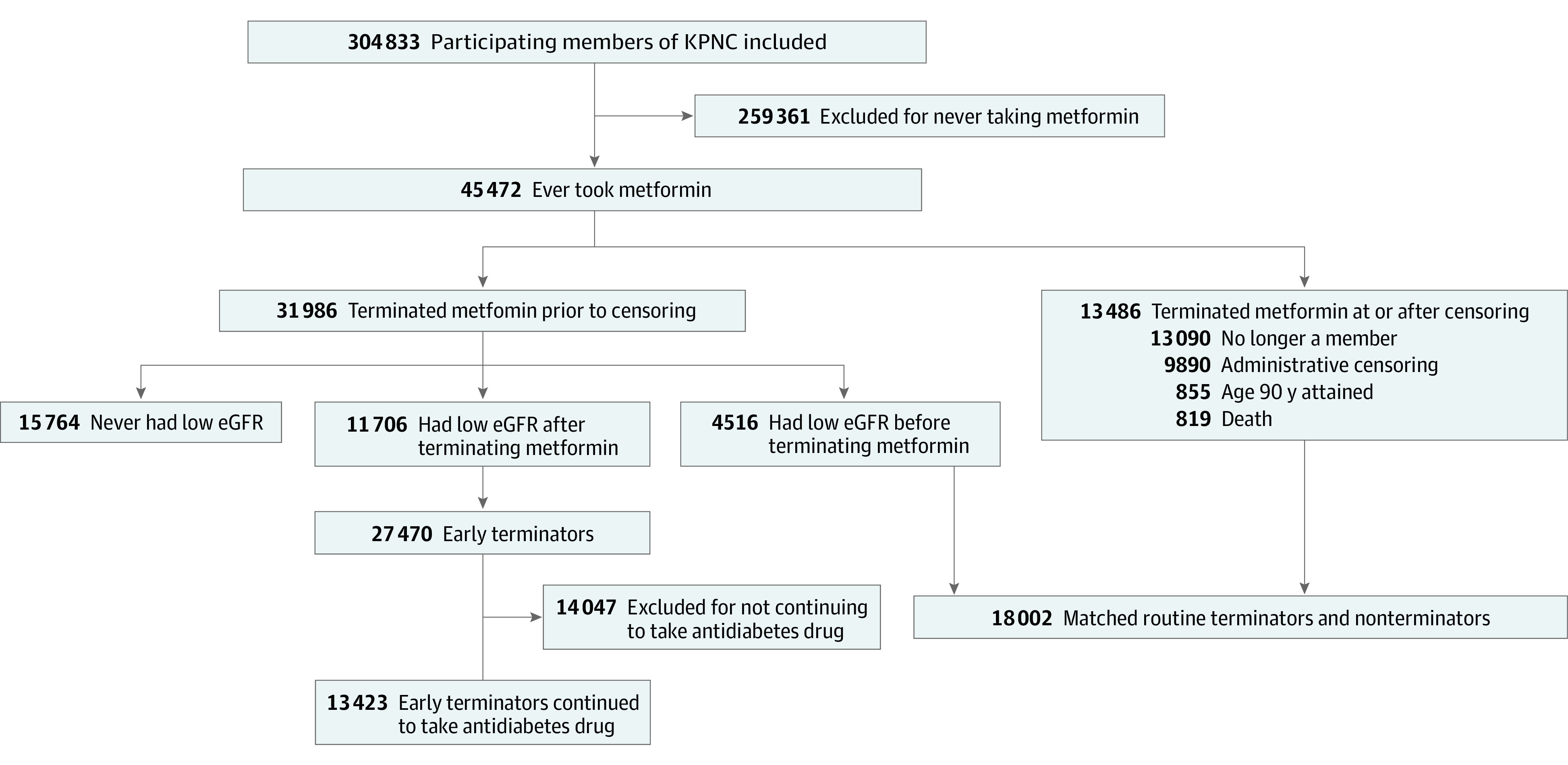
Study Flowchart The flowchart displays inclusions and exclusions for the analytic sample in the Kaiser Permanente Northern California (KPNC) cohort and exposure definition for early terminators and matched routine users. The final sample eligible for matching is shown in the 2 boxes at the bottom. Note that this does not match the sample sizes in Table 1 given that not every early terminator could be matched and some early terminators were matched with other early terminators prior to early termination. eGFR indicates estimated glomerular filtration rate.

The final analytic sample consisted of 12 220 early terminators (5640 women [46.2%]; mean [SD] at start of first metformin prescription, 59.4 [9.0] years; 1642 Asian [13.4%], 1004 Black [8.2%], 1819 Hispanic [14.9%], and 7663 White [62.7%]) and 29 126 routine users (13 582 women [46.6%]; mean [SD] age at start of first metformin prescription, 61.1 [8.9] years; 4674 Asian [16.0%], 2131 Black [7.3%], 3832 Hispanic [13.2%], and 18 286 White [62.8%]) (Table 1). Figure 2 shows the number of individuals meeting inclusion and exclusion criteria. Table 1 gives additional demographic information. At the age of matching among 29 126 matched pairs, 18 686 pairs (64.2%) had an HbA_1c_ level within 0.1 percentage points of each other, 17 563 pairs (60.3%) had ages within 1 year of each other, and 18 064 pairs (62.0%) had a duration with diabetes within 1 year of each other (eTables 1 and 2 in Supplement 1). There were 724 routine users and 281 early terminators who reached age 90 years, 5172 routine users and 3434 early terminators who died, and 6286 routine users and 2181 early terminators who left the Kaiser system. See eTables 3 through 6 in Supplement 1 for the final analytic samples for sensitivity analyses. Cox models were stratified by age at metformin initiation and gender. Proportional hazard assumptions were met at a significance threshold of .05.

**Table 1.** Characteristics of the Analytic Sample

Characteristic	Metformin users, No. (%)^a^								
	Men and women			Men			Women		
	Overall (N = 41 346)	Early terminators (n = 12 220)	Routine users (n = 29 126)	Overall (n = 22 124 [53.5%])	Early terminators (n = 6580)	Routine users (n = 15 544)	Overall (n = 19 222 [46.5%)	Early terminators (n = 5640)	Routine users (n = 13 582)
Age at start of first metformin prescription, mean (SD), y	60.6 (9.0)	59.4 (9.0)	61.1 (8.9)	60.3 (8.7)	59.1 (8.8)	60.8 (8.7)	60.9 (9.2)	59.7 (9.3)	61.4 (9.1)
HbA_1c_ level, mean (SD), %	7.8 (1.3)	8 (1.4)	7.7 (1.3)	7.8 (1.3)	7.9 (1.4)	7.7 (1.3)	7.8 (1.3)	8 (1.4)	7.7 (1.3)
Race and ethnicity									
Asian	6316 (15.3)	1642 (13.4)	4674 (16.0)	3524 (15.9)	943 (14.3)	2581 (16.6)	2792 (14.5)	699 (12.4)	2093 (15.4)
Black	3135 (7.6)	1004 (8.2)	2131 (7.3)	3014 (13.6)	969 (14.7)	2045 (13.2)	12 025 (62.6)	3534 (62.7)	8491 (62.5)
Hispanic	5651 (13.7)	1819 (14.9)	3832 (13.2)	1495 (6.8)	487 (7.4)	1008 (6.5)	2637 (13.7)	850 (15.1)	1787 (13.2)
White	25 949 (62.8)	7663 (62.7)	18 286 (62.8)	13 924 (62.9)	4129 (62.8)	9795 (63.0)	1640 (8.5)	517 (9.2)	1123 (8.3)
Unreported or other^b^	295 (0.7)	92 (0.8)	203 (0.7)	167 (0.8)	52 (0.8)	115 (0.7)	128 (0.7)	40 (0.7)	88 (0.6)
Education									
≥College	13 879 (33.6)	3799 (31.1)	10 080 (34.6)	8357 (37.8)	2301 (35)	6056 (39)	2092 (10.9)	657 (11.6)	1435 (10.6)
High school	19 549 (47.3)	5924 (48.5)	13 625 (46.8)	9940 (44.9)	3044 (46.3)	6896 (44.4)	5522 (28.7)	1498 (26.6)	4024 (29.6)
<High school	4100 (9.9)	1317 (10.8)	2783 (9.6)	2008 (9.1)	660 (10.0)	1348 (8.7)	9609 (50.0)	2880 (51.1)	6729 (49.5)
Other	229 (0.6)	60 (0.5)	169 (0.6)	106 (0.5)	30 (0.5)	76 (0.5)	123 (0.6)	30 (0.5)	93 (0.7)
Missing data	3589 (8.7)	1120 (9.2)	2469 (8.5)	2008 (9.1)	660 (10.0)	1348 (8.7)	1876 (9.8)	575 (10.2)	1301 (9.6)
Nativity									
Not US born	8687 (21.0)	2390 (19.6)	6297 (21.6)	4741 (21.4)	1337 (20.3)	3404 (21.9)	3946 (20.5)	1053 (18.7)	2893 (21.3)
US born	30 717 (74.3)	9219 (75.4)	21 498 (73.8)	16 082 (72.7)	4828 (73.4)	11 254 (72.4)	14 635 (76.1)	4391 (77.9)	10 244 (75.4)
Country of birth unknown to participant	131 (0.3)	35 (0.3)	96 (0.3)	104 (0.5)	26 (0.4)	78 (0.5)	27 (0.1)	9 (0.2)	18 (0.1)
Missing data	1811 (4.4)	576 (4.7)	1235 (4.2)	1197 (5.4)	389 (5.9)	808 (5.2)	614 (3.2)	187 (3.3)	427 (3.1)
Baseline disease history									
Cardiovascular	11 752 (28.4)	4036 (33.0)	7716 (26.5)	7470 (33.8)	2567 (39.0)	4903 (31.5)	4282 (22.3)	1469 (26.0)	2813 (20.7)
Cancer history	8511 (20.6)	2450 (20.0)	6061 (20.8)	4789 (21.6)	1389 (21.1)	3400 (21.9)	3722 (19.4)	1061 (18.8)	2661 (19.6)
Diabetes	41 346 (100)	12 220 (100)	29 126 (100)	22 124 (100)	6580 (100)	15 544 (100)	19 222 (100)	5640 (100)	13 582 (100)
Time since diabetes diagnosis at baseline, mean (SD), y	6.6 (5.0)	7.3 (5.1)	6.3 (4.9)	7 (5.1)	7.7 (5.2)	6.6 (5.0)	6.2 (4.8)	6.8 (5.0)	5.9 (4.7)
Metformin was first diabetes prescription	28 880 (69.8)	8737 (71.5)	20 143 (69.2)	15 644 (70.7)	4822 (73.3)	10 822 (69.6)	13 236 (68.9)	3915 (69.4)	9321 (68.6)
High creatinine at initiation of metformin^c^	4456 (10.8)	1183 (9.7)	3273 (11.2)	2975 (13.4)	819 (12.4)	2156 (13.9)	1481 (7.7)	364 (6.5)	1117 (8.2)
Low eGFR at initiation of metformin^d^	1531 (3.7)	196 (1.6)	1335 (4.6)	675 (3.1)	95 (1.4)	580 (3.7)	856 (4.5)	101 (1.8)	755 (5.6)

Abbreviations: eGFR, estimated glomerular filtration rate; HbA_1c_, hemoglobin A_1c_.

^a^ Baseline refers to the time of metformin termination or corresponding time for matched routine users. Note that this does not match the sample sizes in Figure 2 because not every early terminator could be matched and some early terminators were matched with other early terminators prior to early termination.

^b^ See the eMethods in Supplement 1 for additional information on racial and ethnic categories.

^c^ Gender-specific cutoffs of 1.4 mg/dL (124 μmol/L) or greater for women and 1.5 mg/dL (133 μmol/L) or greater for men were used.

^d^ A cutoff of 45 mL/min/1.73 m^2^ was used.

In minimally adjusted models, early terminators had 1.21 times the hazard of dementia diagnosis compared with routine users (HR, 1.21; 95% CI, 1.15-1.28). In a fully adjusted model, early terminators had 1.21 times the hazard of dementia diagnosis compared with routine users (HR, 1.21; 95% CI, 1.12-1.30) (Table 2; eFigure 2 and eTable 7 in Supplement 1). For the purposes of comparisons with studies of metformin initiation, the reciprocal of the fully adjusted HR was 0.83 (95% CI, 0.76-0.90). When estimated with AFT models, this association translated to an accelerated time to dementia of 0.89 (95% CI, 0.83-0.96) in the minimally adjusted model and 0.89 (95% CI, 0.82-0.97) in the fully adjusted model with early termination compared with routine use. Sensitivity analyses yielded similar results to the main analysis. Table 2 provides a summary of main results and sensitivity analyses.

**Table 2.** Main Results and Sensitivity Analyses

Analysis	Model	Dementia, HR (95% CI)^a^	Accelerated time to dementia from AFT model (95% CI)
Main	Minimally adjusted	1.21 (1.15-1.28)	0.89 (0.83-0.96)
	Fully adjusted	1.21 (1.12-1.30)	0.89 (0.82-0.97)
Sensitivity analysis			
Creatinine^b^	Minimally adjusted	1.22 (1.15-1.29)	0.89 (0.82-0.96)
	Fully adjusted	1.21 (1.12-1.30)	0.89 (0.81-0.98)
High adherence^c^	Minimally adjusted	1.21 (1.14-1.28)	0.89 (0.82-0.96)
	Fully adjusted	1.21 (1.12-1.29)	0.89 (0.82-0.98)
Terminators with <2 y^d^	Minimally adjusted	1.34 (1.26-1.42)	0.87 (0.80-0.95)
	Fully adjusted	1.28 (1.17-1.38)	0.90 (0.81-1.00)
Tight filter^e^	Minimally adjusted	1.30 (1.23-1.38)	0.87 (0.82-0.93)
	Fully adjusted	1.27(1.17-1.36)	0.89 (0.82-0.95)

Abbreviations: AFT, accelerated failure time; HR, hazard ratio.

^a^ Point estimates and 95% CIs are given for HRs and accelerated time to dementia for terminating metformin early. Minimally adjusted HRs are adjusted only for matching variables, and fully adjusted HRs are adjusted for demographic characteristics and comorbidities at the time of metformin initiation.

^b^ Sensitivity analysis using high creatinine level (gender-specific cutoffs of ≥1.4 mg/dL [124 μmol/L] for women and ≥1.5 mg/dL [133 μmol/L] for men were used) instead of low estimated glomerular filtration rate (a cutoff of 45 mL/min/1.73 m^2^ was used) as the criterion for abnormal kidney markers.

^c^ Sensitivity analysis using an analytic sample limited to participants with high adherence (>80%).

^d^ Sensitivity analysis using an analytic sample limited to early terminators with less than 2 years of follow-up.

^e^ Sensitivity analysis using an analytic sample with tighter matching criteria.

**Figure 3.**
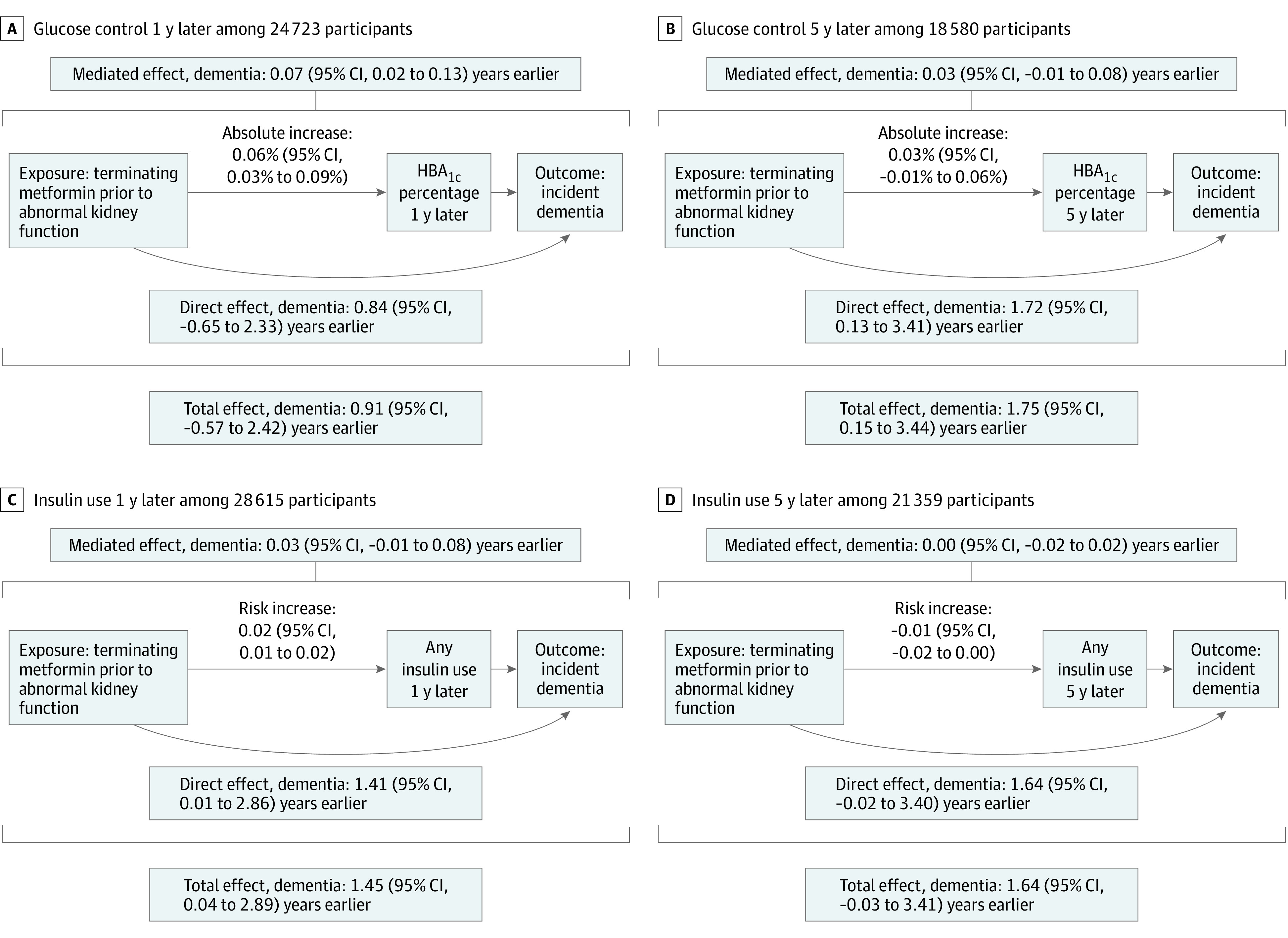
Mediation Analysis Results Sample sizes differed across mediation analyses due to data availability for mediators.

Figure 3 presents mediation analyses. Exposure-mediator interactions were not significant for any models and were not included. Total estimated acceleration of dementia diagnosis ranged from 0.91 years (95% CI, −0.57 to 2.42 years) for HbA_1c_ level at 1 year to 1.75 years (95% CI, 0.15 to 3.44 years) for HbA_1c_ level at 5 years. The mediated acceleration contributed by changes in HbA_1c_ level or insulin use ranged from no contribution (0.00 years (95% CI, −0.02 to 0.02 years) for insulin use at 5 years to 0.07 years (95% CI, 0.02 to 0.13 years) for HbA_1c_ level at 1 year, suggesting that a small fraction of the acceleration of dementia diagnosis could be attributed to measured changes in HbA_1c_ level or insulin use. Additional estimates and 95% CIs are presented in Figure 3.

## Discussion

This cohort study found that terminating metformin treatment was associated with increased dementia incidence in a diverse cohort of older adults. Most of this association was not accounted for by increases in HbA_1c_ levels or insulin use 1 or 5 years after cessation of treatment with metformin.

This work has several important implications. First, findings corroborate the largely consistent evidence from other observational studies showing an association between metformin use and lower dementia incidence. A major advantage of our design was that it may have reduced potential for confounding by indication or cohort effects present in initiation designs given that all individuals in our analyses were metformin users. In addition, the very large sample size and long follow-up provided fairly precise estimates. Previously meta-analyzed results^10^ indicated that people with diabetes who receive metformin had 0.76 times the hazard of Alzheimer disease compared with people with diabetes not receiving metformin (HR, 0.76; 95% CI, 0.60-0.97). This meta-analyzed result included control groups of patients with diabetes receiving sulphonylurea therapy or no drug therapy or any patients with diabetes not receiving metformin. A prior study^10,37^ found an HR of 0.82 (95% CI, 0.52-1.28) comparing metformin with sulphonylurea therapy. Thus, our result of 0.83 times the hazard of dementia with routine use compared with early termination (reciprocal of the fully adjusted HR, 0.83; 95% CI, 0.76-0.90) is consistent with the literature on metformin treatment and dementia incidence.

Second, these results may have potential implications for diabetes treatment in late life. For individuals with diabetes at particularly high risk of dementia, such as carriers of *APOE* ε4 or individuals with a family history of dementia, it may be particularly beneficial to find ways to manage or mitigate gastrointestinal adverse effects (eg, switching to slower-release formulations of metformin or taking the medication with food in the evening)^20^ instead of replacing metformin with other agents given that participants in this study remained on antidiabetes drugs after early termination with metformin. Formally evaluating the heterogeneity of the metformin estimate across known risk factors for dementia is an important extension of our work. Given considerable interest in drug repurposing for dementia, further confirmatory work would be required to extrapolate to prediabetic or nondiabetic populations.^38,39^

Our analysis emulates an intent-to-treat analysis: we did not compare early terminators only with individuals who stayed on metformin indefinitely or until censoring, nor did we only compare early terminators with individuals who terminated due to kidney dysfunction. Such separate comparisons would have the same biases that as-treated analyses have^40^ because, assuming that discontinuing metformin is not associated with dementia, early terminators would be expected to have increased risk of dementia compared with those who did not terminate simply because some early terminators will develop kidney dysfunction. Similarly, early terminators as a group would be expected to have reduced risk of dementia compared with individuals who terminated due to signs of kidney dysfunction because not all early terminators will develop kidney dysfunction. Thus, both such comparisons would be biased metformin was not associated with dementia.

### Strengths

This study has several strengths. First, while we did not use a standard quasiexperimental design,^41^ we leveraged the fact that gastrointestinal adverse effects are a common reason for discontinuation of metformin but are likely unrelated to diabetes progression or dementia risk. Second, all individuals were metformin initiators, mitigating the potential for cohort effects and confounding by indication. Additionally, beginning follow-up at the age of early termination of metformin use prevents immortal person-time bias.^17^

### Limitations

This study also has several limitations. First, dementia diagnosis was obtained based on medical records, and recording of such diagnoses likely follows onset of pathology. Second, this was a complete case analysis; thus, the main analysis and mediation samples differed in size, although estimates for total effect sizes were consistent in both analyses. Third, we did not evaluate numerous and relevant potential axes of heterogeneity, such as race, ethnicity, or duration of metformin use. Our reported associations are means over the distributions of the history of metformin use and thus would not be appropriate for clinicians for specific patient recommendations. Fourth, as stated previously, data on the precise reason for termination of metformin were not available. Metformin initiation is contraindicated in severe liver disease and heart failure. However, because metformin is beneficial for people with diabetes with congestive heart failure, chronic liver disease, or reduced kidney function (eGFR, 45-60 mL/min/1.73 m^2^),^42^ we did not consider these reasons for termination in routine users. It is possible that our characterization of early metformin terminators may have misclassified some individuals. However, in a sensitivity analysis that examined only individuals who terminated use within the first 2 years of therapy, we had comparable results. In this analysis, we would be assuming that very early termination was less likely to be due to kidney dysfunction. Fifth, due to data limitations, we could not address every potential source of bias, such as deprescribing to improve quality of life in individuals with frailty, and we did not consider time-updated mediator outcome confounding. However, these are different confounding biases than would be present in studies of treatment initiation that indicate a comparable benefit of metformin. If censoring due to reaching age 90 years, death, or KPNC membership gap and another risk factor associated with dementia both affect treatment and outcome status, effect estimates could be biased. Early terminators were more likely to die during follow-up, so expected bias would likely be to reduce the estimated effect size in the association of early termination with dementia risk. Relatedly, to ensure privacy, all data related to time were provided with age as the time scale as opposed to calendar time. However, it is possible that calendar time was associated with non-kidney-disease–related metformin cessation. Sixth, the applicability of these results relies on the assumption that individuals experiencing significant gastrointestinal or other adverse effects were not also more likely to experience diabetes progression for other reasons. Factors such as high dietary carbohydrate consumption could be associated with adverse effect prevalence and diabetes progression. However, confounders of metformin discontinuation due to adverse effects were likely different than those for metformin initiation, and our estimates for terminating metformin were similar to estimates of initiating metformin.

## Conclusions

In this cohort study of metformin users, terminating metformin treatment was associated with increased dementia incidence, corroborating prior observational research that initiating metformin was associated with reduced risk of dementia. This finding has important implications for the clinical management of diabetes.

## Notes

Supplement 1.
eMethods.
**eFigure 1.** Conceptual Model **eFigure 2.** Dementia-Free Probability Curve **eTable 1.** Match Quality Table for Main Analysis **eTable 2.** Match Quality Table for Sensitivity Analysis With Tighter Matches **eTable 3.** Characteristics of the Creatinine-Based Analytic Sample, Overall and for Early and Nonearly Terminators **eTable 4.** Characteristics of the Analytic Sample Limited to High-Adherence Participants, Overall and for Early and Nonearly Terminators **eTable 5.** Characteristics of the Analytic Sample Using Tighter Matching Criteria, Overall and for Early and Nonearly Terminators **eTable 6.** Characteristics of the Analytic Sample Limited to Early Terminators With <2 y Follow-Up, Overall and for Early and Nonearly Terminators **eTable 7.** Exponentiated Coeﬃcients for All Models

Supplement 2. **eTable 8.** Electronic Health Record Data **eTable 9.** Prescriptions

Supplement 3.
Data Sharing Statement
