# Supplementary material for: Metformin Cessation and Dementia Incidence
Source: JAMA Netw Open. 2023 Oct 25;6(10):e2339723. doi: 10.1001/jamanetworkopen.2023.39723 (PMC10600586; doi:10.1001/jamanetworkopen.2023.39723)
Supplement: Supplement 1. — eMethods. eFigure 1. Conceptual Model eFigure 2. Dementia-Free Probability Curve eTable 1. Match Quality Table for Main Analysis eTable 2. Match Quality Table for Sensitivity Analysis With Tighter Matches eTable 3. Characteristics of the Creatinine-Based Analytic Sample, Overall and for Early and Nonearly Terminators eTable 4. Characteristics of the Analytic Sample Limited to High-Adherence Participants, Overall and for Early and Nonearly Terminators eTable 5. Characteristics of the Analytic Sample Using Tighter Matching Criteria, Overall and for Early and Nonearly Terminators eTable 6. Characteristics of the Analytic Sample Limited to Early Terminators With <2 y Follow-Up, Overall and for Early and Nonearly Terminators eTable 7. Exponentiated Coeﬃcients for All Models [file jamanetwopen-e2339723-s001.pdf]

Supplemental Online Content

Zimmerman SC, Ferguson EL, Choudhary V, et al. Metformin cessation and dementia incidence. *JAMA Netw Open*. 2023;6(10):e2339723. doi:10.1001/jamanetworkopen.2023.39723

eMethods.

eFigure 1. Conceptual Model

eFigure 2. Dementia-Free Probability Curve

eTable 1. Match Quality Table for Main Analysis

eTable 2. Match Quality Table for Sensitivity Analysis With Tighter Matches

eTable 3. Characteristics of the Creatinine-Based Analytic Sample, Overall and for Early and Nonearly Terminators

eTable 4. Characteristics of the Analytic Sample Limited to High-Adherence Participants, Overall and for Early and Nonearly Terminators

eTable 5. Characteristics of the Analytic Sample Using Tighter Matching Criteria, Overall and for Early and Nonearly Terminators

eTable 6. Characteristics of the Analytic Sample Limited to Early Terminators With <2 y Follow-Up, Overall and for Early and Nonearly Terminators

eTable 7. Exponentiated Coefficients for All Models

This supplemental material has been provided by the authors to give readers additional information about their work.

© 2023 Zimmerman SC et al. *JAMA Network Open.*

Methods Supplement

Diabetes Registry

Individuals meeting any one of four criteria were considered to have diabetes and included in the registry: (1) a single, principal inpatient diabetes mellitus (DM) diagnosis; (2) two or more outpatient diabetes diagnoses (ICD-9 and ICD-10 codes) during the previous 5 years; (3) two or more abnormal outpatient lab test results during the previous two years (i.e., HbA1c ≥ 6.5%, fasting glucose ≥ 126 mg/dL, random glucose or postload glucose ≥ 200 mg/dL); or (4) at least one dispensing for a medication used to treat DM (insulin, oral hypoglycemic agent, antihyperglycemic agents). Individuals who use metformin or thiazolidinediones and have certain diagnoses (i.e., polycystic ovary syndrome) during the past 2 years, but did not meet any of the other diabetes criteria were excluded from the registry. Patients with evidence of gestational diabetes are also excluded from the registry.

Diagnosic Records

We determined diabetes status (type 1, type 2, or neither) the from the KPNC Diabetes Registry. The registry has records dating back to 1978 for any encounter type, allowing for ascertainment of diabetes diagnosis beyond what would be available in electronic health records. Additional information on the registry is available in the Supplement and citation below. We defined clinical diagnoses of cardiovascular disease, cancer, kidney disease, and dementia in terms of groups of International Classification of Diseases, Ninth Revision (ICD-9) diagnostic codes for January 1996 to September 2015 and International Classification of Diseases, Tenth Revision (ICD-10) diagnostic codes after September 2015. The outcome measure of all-cause dementia diagnosis was defined as first diagnosis of any of the following: Alzheimer's disease, vascular dementia, non-specific dementia, dementia with Lewy bodies, and Parkinson's disease. All diagnostic codes are given in DiagnosticCodes.xlsx.

Citation: Moffet, H.H., Adler, N., Schillinger, D., Ahmed, A.T., Laraia, B., Selby, J.V., Neugebauer, R., Liu, J.Y., Parker, M.M., Warton, M. and Moffet, H.H., 2007. The

© 2023 Zimmerman SC et al. *JAMA Network Open.*

Diabetes Study of Northern California (DISTANCE): Objectives and design of a survey follow-up study of social health disparities in a managed care population.

Laboratory Records

Estimated glomerular filtration rate and serum creatinine test results were extracted from KPNC lab records. We classified low eGFR values (less than 45 mL/min/1.73 m2) as suggestive of impaired kidney function. In a sensitivity analysis, we classified high creatinine measures (at or over 1.4 mg/dL for women or 1.5 mg/dL for men) as suggestive of impaired kidney function.

Prescription Records

Prescriptions of metformin, insulins, and other antidiabetes drugs were extracted from KPNC pharmacy dispensing records by generic name. Periods of continuous medication use were calculated without respect to dosage. Each prescription record included a start date and a number of days' supply. Prescription end dates were calculated by adding the days' supply to the start date, and prescriptions were collapsed into contiguous periods if the gap between the end of a prescription and the start of the next prescription was 182 or fewer days ("persistence requirement"). For collapsed prescriptions, adherence was calculated as the number of days of medication supply divided by the remaining days on the prescription. A new prescription record was created if adherence fell below 0.8. For combination medications, separate records were included for each component drug.

Details on prescriptions are given in DiagnosticCodes.xlsx.

Sociodemographic Variables

Gender was extracted from KPNC health plan membership databases. Other sociodemographic covariates (race, ethnicity, educational attainment, nativity, parental nativity, and survey language) were self-reported in the CMHS or RPGEH surveys.

Self-reported race and ethnicity categories were collapsed into the following categories: Black, Hispanic/Latino, Asian, White and other/uncertain. The CMHS and RPGEH surveys asked participants to endorse specific racial/ethnic identities (RPGEH: "What best describes your race or ethnicity? Mark all groups that apply to you": AfricanAmerican, African, Afro-Caribbean, Mexican, Central/South American, Puerto

© 2023 Zimmerman SC et al. *JAMA Network Open.*

Rican, Cuban, Other Latino/Hispanic, South Asian (Indian, Pakistani, etc.), Chinese, Japanese, Korean, Filipino, Vietnamese, Other Southeast Asian (Cambodian, Laotian, etc.), Native Hawaiian, Samoan, Other Pacific Islander, Native American Indian or Alaska Native, White or European-American, Middle Eastern, Ashkenazi Jewish, Other (please specify), and Don't know; CMHS: White-European, White-Middle Eastern, Mexican Central South American or other Hispanic, Black or African American, American Indian or Alaskan Native, Asian Indian, Chinese, Filipino, Japanese (includes Okinawan), Korean, Vietnamese or other Southeast Asian, Other Asian (Print race/ethnicity), Native Hawaiian, Guamanian or Chamorro, Samoan, Other Pacific Islander (Print race/ethnicity), Some other race/ethnicity (Print race/ethnicity)). Participants were instructed to endorse all groups that applied. For individuals who endorsed more than one race or ethnicity, these indicators were collapsed using an algorithm to assign one adjudicated race/ethnicity with the following prioritization order: Black, Hispanic, Asian, Native American (with no other racial/ethnic group endorsement). If a participant did not endorse any ethnicities on their survey, race/ethnicity was derived from the KPNC health plan databases. The algorithm was validated by comparing against several sources, including the KPNC virtual data warehouse (VDW), Utility for Care Data Analysis (UCDA; a Kaiser division that collects and cleans demographic data on Kaiser membership), and principal components from genetic data (available for ~25% of participants). The majority of the full RPGEH cohort had race/ethnicity values from the survey that matched these other sources and genetic data when available. For the small percentage (~2%) that did not match, surveys were checked by hand (to account for possible scanning errors), and race/ethnicity endorsement(s) were overwritten with VDW data when errors were found.

Diagnostic Codes

Supplemental Tables and Figures

© 2023 Zimmerman SC et al. *JAMA Network Open.*

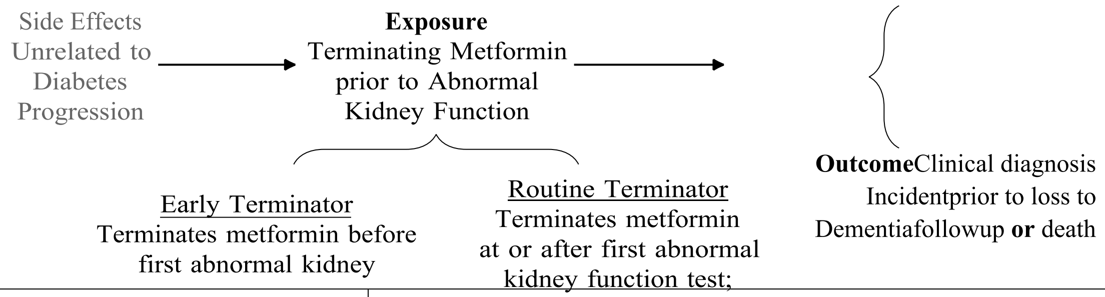

Diagnosis Name	ICD-9 Diagnosic Codes
Alzheimer's Disease	331.0 (Alzheimer's disease)
Vascular Dementia	290.4x (Vascular dementia)
Nonspecific Dementia	290.0, 290.1x, 290.2x, 290.3, 294.2x, 294.8 (Nonspe
Dementia with Lewy Bodies	331.82 (dementia with lewy bodies)
Parksinson's Disease	332.0 (Paralysis agitans) AND 294.1x on same
Hypertension	”362.11 (Hypertensive retinopathy) 401.x-404.xx (Hypertensive Disease) 4

Figure 1: **Conceptual Model.**

The association of early metformin termination with incident dementia (prior to death, end of KPNC membership, or end of follow-up) is evaluated. Early terminators are individuals who terminate metformin prior to an abnormal kidney function test. Early terminators are compared to routine users, individuals who terminate metformin at or after first abnormal kidney function test or do not terminate treatment with metformin.

© 2023 Zimmerman SC et al. *JAMA Network Open.*

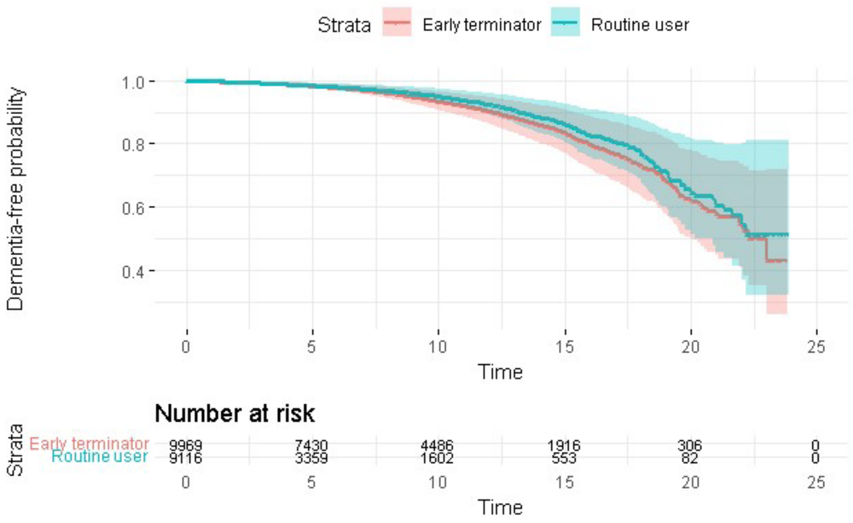

Figure 2: **Dementia-free Probability Curve**.

Based on the fully adjusted model in the main analysis, early terminators show an increased incidence of dementia compared to routine users. Blue line represents routine users and red line indicates early terminators. Shaded areas represent respective 95% confidence intervals.

© 2023 Zimmerman SC et al. *JAMA Network Open.*

Table 1: Match quality table for main analysis

mean	minimum	5th percentile	25th percentile	median	75th percentile	95th percentile	maximum	threshold	percent within						
threshold	RX START AGE	0.17	-5.00	-4.22	-1.86	0.22	2.23	4.33	5.00	1.00	0.60 BL HBA1C	-0.02	-0.50	-0.45	-0.25
-0.03	0.20	0.43	0.50	0.10	0.64 DB DUR	-0.02	-5.00	-4.36	-2.22	-0.01	2.18	4.33	5.00	1.00	0.62

Table 2: Match quality table for sensitivity analysis with tighter matches

	mean	minimum	5th percentile	25th percentile	median	75th percentile	95th percentile	maximum	threshold	percent within threshold					
RX START AGE	0.27	-5.00	-4.04	-1.47	0.28	2.16	4.29	5.00	1.00	0.61 BL HBA1C	0.00	-0.10	-0.10	-0.05	0.00
0.05	0.10	0.10	0.10	1.00 DB DUR	0.00	-1.00	-0.90	-0.48	0.01	0.50	0.90	1.00	1.00	1.00	

© 2023 Zimmerman SC et al. *JAMA Network Open.*

Table 3: Characteristics of the creatinine-based analytic sample, overall and for early and routine terminators

	Overall	Early Terminators	Non-early terminators
n	37802	10757	27045
Age at start of first metformin prescription	60.5 (8.9)	59.3 (9.1)	61 (8.8)
Women	18265 (48.3%)	5336 (49.6%)	12929 (47.8%)
Glycated Hemoglobin (HbA1c, %)	7.8 (1.3)	8 (1.4)	7.7 (1.3)
White race/ethnicity	23588 (62.4%)	6693 (62.2%)	16895 (62.5%)
Asian race/ethnicity	5905 (15.6%)	1476 (13.7%)	4429 (16.4%)
Black race/ethnicity	2792 (7.4%)	855 (7.9%)	1937 (7.2%)
Hispanic race/ethnicity	5249 (13.9%)	1652 (15.4%)	3597 (13.3%)
Unreported or other race/ethnicity	268 (0.7%)	81 (0.8%)	187 (0.7%)
High school education	17923 (47.4%)	5255 (48.9%)	12668 (46.8%)
College education or more	12732 (33.7%)	3322 (30.9%)	9410 (34.8%)
Less than high school education	3669 (9.7%)	1137 (10.6%)	2532 (9.4%)
Other education	224 (0.6%)	62 (0.6%)	162 (0.6%)
Missing education data	3254 (8.6%)	981 (9.1%)	2273 (8.4%)
US Born	27928 (73.9%)	8066 (75%)	19862 (73.4%)
Not US Born	8108 (21.4%)	2156 (20%)	5952 (22%)
Missing country of birth	1642 (4.3%)	503 (4.7%)	1139 (4.2%)
Unknown country of birth	124 (0.3%)	32 (0.3%)	92 (0.3%)
Baseline cardiovascular disease history	10130 (26.8%)	3234 (30.1%)	6896 (25.5%)
Baseline cancer history	7773 (20.6%)	2137 (19.9%)	5636 (20.8%)
Baseline diabetes history	37802 (100%)	10757 (100%)	27045 (100%)
Years since diabetes diagnosis at baseline	6.5 (5)	7.2 (5.2)	6.3 (4.9)
Metformin was first diabetes prescription	26240 (69.4%)	7634 (71%)	18606 (68.8%)
High creatinine at initiation of metformin	3630 (9.6%)	827 (7.7%)	2803 (10.4%)

© 2023 Zimmerman SC et al. *JAMA Network Open.*

Low GFR at initiation of metformin	1512 (4%)	224 (2.1%)	1288 (4.8%)

© 2023 Zimmerman SC et al. *JAMA Network Open.*

Table 4: Characteristics of the analytic sample limited to high-adherence participants, overall and for early and routine terminators

	Overall	Early Terminators	Non-early terminators
n	40240	11803	28437
Age at start of first metformin prescription	60.6 (9)	59.4 (9.1)	61.1 (8.9)
Women	18698 (46.5%)	5447 (46.1%)	13251 (46.6%)
Glycated Hemoglobin (HbA1c, %)	7.8 (1.3)	8 (1.4)	7.7 (1.3)
Asian race/ethnicity	6154 (15.3%)	1582 (13.4%)	4572 (16.1%)
White race/ethnicity	25313 (62.9%)	7420 (62.9%)	17893 (62.9%)
Black race/ethnicity	3030 (7.5%)	971 (8.2%)	2059 (7.2%)
Hispanic race/ethnicity	5457 (13.6%)	1742 (14.8%)	3715 (13.1%)
Unreported or other race/ethnicity	286 (0.7%)	88 (0.7%)	198 (0.7%)
College education or more	13564 (33.7%)	3687 (31.2%)	9877 (34.7%)
High school education	19010 (47.2%)	5717 (48.4%)	13293 (46.7%)
Less than high school education	3956 (9.8%)	1260 (10.7%)	2696 (9.5%)
Missing education data	3484 (8.7%)	1079 (9.1%)	2405 (8.5%)
Other education	226 (0.6%)	60 (0.5%)	166 (0.6%)
Not US Born	8447 (21%)	2294 (19.4%)	6153 (21.6%)
US Born	29912 (74.3%)	8923 (75.6%)	20989 (73.8%)
Unknown country of birth	126 (0.3%)	33 (0.3%)	93 (0.3%)
Missing country of birth	1755 (4.4%)	553 (4.7%)	1202 (4.2%)
Baseline cardiovascular disease history	11464 (28.5%)	3909 (33.1%)	7555 (26.6%)
Baseline cancer history	8309 (20.6%)	2375 (20.1%)	5934 (20.9%)
Baseline diabetes history	40240 (100%)	11803 (100%)	28437 (100%)
Years since diabetes diagnosis at baseline	6.6 (5)	7.3 (5.1)	6.3 (4.9)
Metformin was first diabetes prescription	28066 (69.7%)	8416 (71.3%)	19650 (69.1%)
High creatinine at initiation of metformin	4356 (10.8%)	1154 (9.8%)	3202 (11.3%)

© 2023 Zimmerman SC et al. *JAMA Network Open.*

Low GFR at initiation of metformin	1505 (3.7%)	197 (1.7%)	1308 (4.6%)

© 2023 Zimmerman SC et al. *JAMA Network Open.*

Table 5: Characteristics of the analytic sample using tighter matching criteria, overall and for early and routine terminators

	Overall	Early Terminators	Non-early terminators
n	33347	10572	22775
Age at start of first metformin prescription	60.4 (8.2)	59.7 (8.5)	60.8 (8.1)
Women	15325 (46%)	4819 (45.6%)	10506 (46.1%)
Glycated Hemoglobin (HbA1c, %)	7.6 (1.2)	7.8 (1.3)	7.5 (1.2)
Asian race/ethnicity	5180 (15.5%)	1428 (13.5%)	3752 (16.5%)
White race/ethnicity	21005 (63%)	6683 (63.2%)	14322 (62.9%)
Black race/ethnicity	2463 (7.4%)	845 (8%)	1618 (7.1%)
Hispanic race/ethnicity	4456 (13.4%)	1535 (14.5%)	2921 (12.8%)
Unreported or other race/ethnicity	243 (0.7%)	81 (0.8%)	162 (0.7%)
College education or more	11304 (33.9%)	3282 (31%)	8022 (35.2%)
Less than high school education	3270 (9.8%)	1150 (10.9%)	2120 (9.3%)
Missing education data	2830 (8.5%)	983 (9.3%)	1847 (8.1%)
High school education	15754 (47.2%)	5104 (48.3%)	10650 (46.8%)
Other education	189 (0.6%)	53 (0.5%)	136 (0.6%)
Not US Born	7026 (21.1%)	2079 (19.7%)	4947 (21.7%)
US Born	24750 (74.2%)	7959 (75.3%)	16791 (73.7%)
Unknown country of birth	114 (0.3%)	32 (0.3%)	82 (0.4%)
Missing country of birth	1457 (4.4%)	502 (4.7%)	955 (4.2%)
Baseline cardiovascular disease history	9368 (28.1%)	3474 (32.9%)	5894 (25.9%)
Baseline cancer history	6670 (20%)	2097 (19.8%)	4573 (20.1%)
Baseline diabetes history	33347 (100%)	10572 (100%)	22775 (100%)
Years since diabetes diagnosis at baseline	6.5 (4.5)	6.9 (4.7)	6.2 (4.4)
Metformin was first diabetes prescription	23405 (70.2%)	7605 (71.9%)	15800 (69.4%)
High creatinine at initiation of metformin	3408 (10.2%)	1015 (9.6%)	2393 (10.5%)
Low GFR at initiation of metformin	1028 (3.1%)	145 (1.4%)	883 (3.9%)

© 2023 Zimmerman SC et al. *JAMA Network Open.*

Table 6: Characteristics of the analytic sample limited to early terminators with less than two year follow-up, overall and for early and routine terminators

	Overall	Early Terminators	Non-early terminators
n	30348	7695	22653
Age at start of first metformin prescription	60.8 (9)	59.6 (9.3)	61.2 (8.8)
Women	14613 (48.2%)	3743 (48.6%)	10870 (48%)
Glycated Hemoglobin (HbA1c, %)	7.9 (1.4)	8.2 (1.5)	7.8 (1.3)
White race/ethnicity	18638 (61.4%)	4658 (60.5%)	13980 (61.7%)
Asian race/ethnicity	4914 (16.2%)	1077 (14%)	3837 (16.9%)
Hispanic race/ethnicity	4189 (13.8%)	1208 (15.7%)	2981 (13.2%)
Black race/ethnicity	2391 (7.9%)	692 (9%)	1699 (7.5%)
Unreported or other race/ethnicity	216 (0.7%)	60 (0.8%)	156 (0.7%)
College education or more	10230 (33.7%)	2320 (30.1%)	7910 (34.9%)
Less than high school education	3029 (10%)	879 (11.4%)	2150 (9.5%)
High school education	14272 (47%)	3724 (48.4%)	10548 (46.6%)
Missing education data	2636 (8.7%)	728 (9.5%)	1908 (8.4%)
Other education	181 (0.6%)	44 (0.6%)	137 (0.6%)
US Born	22258 (73.3%)	5727 (74.4%)	16531 (73%)
Not US Born	6679 (22%)	1575 (20.5%)	5104 (22.5%)
Missing country of birth	1307 (4.3%)	368 (4.8%)	939 (4.1%)
Unknown country of birth	104 (0.3%)	25 (0.3%)	79 (0.3%)
Baseline cardiovascular disease history	7344 (24.2%)	2071 (26.9%)	5273 (23.3%)
Baseline cancer history	5601 (18.5%)	1314 (17.1%)	4287 (18.9%)
Baseline diabetes history	30348 (100%)	7695 (100%)	22653 (100%)
Years since diabetes diagnosis at baseline	5 (4.1)	5.4 (4.2)	4.9 (4.1)
Metformin was first diabetes prescription	21777 (71.8%)	5754 (74.8%)	16023 (70.7%)
High creatinine at initiation of metformin	3632 (12%)	887 (11.5%)	2745 (12.1%)
Low GFR at initiation of metformin	1359 (4.5%)	170 (2.2%)	1189 (5.2%)

© 2023 Zimmerman SC et al. *JAMA Network Open.*

Table 7: Exponentiated coefficients for all models.

	Dependentvariable:									
	Crude models					Fully adjusted models				
	Main	Sens. Creatinine	Sens. High Adherence	Sens. Term. less than 2 years	Sens. Tight Filter	Main	Sens. Creatinine	Sens. High Adherence	Sens. Term. less than 2 years	Sens. Tight Filter
Early Termination	1.21***<br>(1.15, 1.28)<br>(1.12, 1.30)	1.22***<br>1.15***	1.21***<br>(1.15, 1.29)<br>(1.12, 1.29)	1.34***<br>(1.14, 1.28)<br>(1.17, 1.38)	1.30***<br>(1.26, 1.42)<br>(1.17, 1.36)	1.21***<br>1.15***	1.21***<br>(1.23, 1.38)	1.21***<br>1.14***	1.28***<br>1.15***	1.27***<br>(1.12, 1.30)
Age	1.15***1.16***1.15***<br>(1.13, 1.16)<br>(1.14, 1.17)	1.00**<br>**<br>(1.13, 1.16)	(1.14, 1.17)<br>(1.13, 1.17)	1.15***1.14***<br>(1.13, 1.16)<br>(1.13, 1.16)	(1.13, 1.17)	1.00**<br>*	(1.13, 1.16)	1.00*** 1.15***	1.00***	(1.13, 1.16)
Age Squared	(1.00, 1.00)<br>(1.00, 1.00)	1.00***1.00***<br>(1.00, 1.00)<br>(1.00, 1.00)	(1.00, 1.00)<br>(1.00, 1.00)	1.00***<br>(1.00, 1.00)	1.00***1.00*1.00**<br>(1.00, 1.00)		(1.00, 1.00)			(1.00, 1.00)
HbA1c (Mean of Last 3)	1.02<br>1.03<br>(0.99, 1.05)<br>(0.99, 1.07)	1.04<br>1.02<br>(1.00, 1.07)<br>(0.98, 1.07)	1.02<br>1.04<br>(0.99, 1.05)<br>(0.99, 1.08)	1.03<br>(0.99, 1.06)	1.02<br>(0.99, 1.06)	1.48***<br>1.24**	1.04<br>(1.00, 1.07)	1.41***<br>1.22**	1.44***<br>1.21*	1.03<br>(0.99, 1.07)
Metformin first Diabetes Drug	1.03<br>1.05<br>(0.94, 1.12)<br>(0.95, 1.15)	1.02<br>0.99<br>(0.92, 1.12)<br>(0.86, 1.13)	1.04<br>1.04<br>(0.95, 1.13)<br>(0.93, 1.16)	1.00<br>(0.88, 1.12)	1.05<br>(0.95, 1.15)	1.38***	1.04<br>(0.94, 1.14)	1.36***	*<br>1.28***	1.03<br>(0.92, 1.14)
Black1.41***1.52***					(1.29, 1.75)	1.23***	(1.27, 1.69)<br>(1.19, 1.62)	1.21***	1.24**	(1.18, 1.64)<br>(1.18, 1.69)
Hispanic1.22**1.29***					(1.10, 1.48)		(1.06, 1.41)<br>(1.04, 1.40)			(1.03, 1.41)<br>(0.99, 1.43)
Other/uncertain/missing race					1.12<br>(0.47, 1.78)	0.77***	1.51<br>1.45<br>(0.91, 2.11)<br>(0.84, 2.05)	–<br>0.78***	–<br>0.76***	1.59<br>1.76<br>(0.98, 2.21)<br>(1.11, 2.41)
White1.35***1.41***							(1.23, 1.54)<br>(1.09, 1.47)(1.24,	*<br>1.57)	1.22**<br>(1.19, 1.51)	(1.20, 1.51)
High School Education							1.02<br>1.03 1.03<br>(0.93, 1.11)<br>(0.91, 1.14)(0.93,	1.13)	1.03<br>(0.93, 1.13)	1.02<br>(0.93, 1.11)
Less than High School Education1.22***1.23***							(1.10, 1.36)<br>(1.07, 1.40)(1.09,	1.37)	(1.08, 1.37)	(1.07, 1.34)
Other Education						1.14*<br>0.92<br>(0.80, 1.79)	1.30<br>1.45<br>(0.83, 1.84)	**<br>(0.67, 1.68)	1.33<br>(0.22, 1.62)	1.17<br>(0.94, 1.96)
Unknown if USA Born						0.91<br>(0.03, 1.80)	0.68<br>(−0.34, 1.69)	0.82<br>(0.12, 1.76)	0.73<br>(0.39, 1.86)	0.99<br>(0.05, 1.92)
USA Born0.78***0.79***						(0.63, 0.92)	(0.63, 0.94)	(0.63, 0.92)	(0.58, 0.95)	(0.63, 0.94)
Unknown if Mother USA Born						1.13<br>(0.52, 1.73)	1.32<br>(0.65, 1.98)	1.06<br>(0.43, 1.69)	1.18<br>(0.41, 1.95)	1.31<br>(0.66, 1.97)
Mother USA Born						1.11<br>(0.97, 1.25)	1.09<br>(0.93, 1.24)	(0.99, 1.28)	1.141.08<br>(1.05, 1.40)	(0.93, 1.23)
Unknown if Father USA Born						1.22<br>(0.85, 1.60)	1.02<br>(0.57, 1.47)	1.29<br>(0.92, 1.66)	1.12<br>(0.68, 1.56)	1.00<br>(0.57, 1.44)
Father USA Born						0.97<br>(0.84, 1.11)	0.98<br>(0.84, 1.13)	0.97<br>(0.84, 1.11)	0.95<br>(0.78, 1.11)	1.00<br>(0.86, 1.14)
English Survey Language						0.97<br>(0.54, 1.41)	0.99<br>(0.54, 1.44)	0.98<br>(0.54, 1.42)	0.88<br>(0.38, 1.39)	0.88<br>(0.44, 1.31)
Spanish Survey Language						0.69<br>(0.08, 1.29)	0.68<br>(0.05, 1.31)	0.71<br>(0.10, 1.32)	0.67<br>(0.04, 1.38)	0.67<br>(0.03, 1.31)
Baseline Hypertension1.09						(0.99, 1.28)	(0.94, 1.24)	1.18<br>(1.03, 1.32)	1.09<br>(0.93, 1.25)	1.09<br>(0.93, 1.24)
LDL (Mean of Last 3)						1.00<br>(1.00, 1.00)	1.00<br>(1.00, 1.00)	1.00<br>(1.00, 1.00)	1.00<br>(1.00, 1.00)	1.00<br>(1.00, 1.00)

© 2023 Zimmerman SC et al. *JAMA Network Open*.

Rx for Antilipemic						0.95	0.92	0.96	0.95	0.98
						(0.85, 1.04)	(0.82, 1.02)	(0.86, 1.05)	(0.84, 1.06)	(0.88, 1.08)
Count of Rx Non-Metformin Oral Hypoglycemic Agents Categories						0.98	0.98 **	0.97	1.06	1.00
						(0.90, 1.06)<br>(0.92, 1.09)		(0.89, 1.07)	(0.89, 1.05)	(0.94, 1.17)
Rx Insulin						1.01<br>0.94	1.13**	1.07	1.03	1.08
						(0.88, 1.14)<br>(0.80, 1.08)	1.27***	(0.93, 1.21)	(0.90, 1.16)	(0.90, 1.26)
Cancer History						0.94	0.90	0.94	0.91	0.97
						(0.84, 1.04)	(0.79, 1.00)	(0.84, 1.04)	(0.78, 1.04)	(0.86, 1.08)
CVD History							1.15***1.15***		1.15**	1.17***
						(1.06, 1.24)	(1.03, 1.23)	(1.05, 1.24)	(1.03, 1.27)	(1.07, 1.27)
Count of Prior CVD Event Categories							1.28***1.28***		1.25***	1.35***
						(1.20, 1.36)	(1.19, 1.36)	(1.20, 1.36)	(1.14, 1.37)	(1.26, 1.43)
History of Low GFR at BL						1.29**		1.25*	1.28*	1.23
						(1.07, 1.51)		(1.02, 1.48)	(1.02, 1.54)	(0.94, 1.52)
History of High Creatinine at Baseline							1.11			
							(0.96, 1.25)			
Baseline Diabetes Duration	1.02***	1.03***	1.02***	1.03***	1.02***	1.02***	1.02***	1.02***	1.03***	1.02***
	(1.02, 1.03)	(1.02, 1.04)	(1.01, 1.03)	(1.02, 1.04)	(1.01, 1.03)	(1.01, 1.03)	(1.01, 1.03)	(1.01, 1.03)	(1.01, 1.04)	(1.01, 1.04)
Baseline Hypertension Duration						1.01	1.01	1.00	1.00	1.00
						(0.99, 1.02)	(0.99, 1.02)	(0.99, 1.02)	(0.99, 1.02)	(0.99, 1.02)
Observations	41,346	37,802	40,240	30,348	33,347	34,211	31,265	33,332	24,730	27,776
R^2	0.02	0.02	0.02	0.01	0.02	0.02	0.03	0.02	0.01	0.03
Note:										*p<0.1; **p<0.05; ***p<0.01

© 2023 Zimmerman SC et al. *JAMA Network Open.*
