# Supplementary material for: Metformin Cessation and Dementia Incidence
Source: JAMA Netw Open. 2023 Oct 25;6(10):e2339723. doi: 10.1001/jamanetworkopen.2023.39723 (PMC10600586; doi:10.1001/jamanetworkopen.2023.39723)
Supplement: Supplement 3. — Data Sharing Statement [file jamanetwopen-e2339723-s003.pdf]

Data Sharing Statement

Zimmerman. Metformin Cessation and Dementia Incidence. *JAMA Netw Open*. Published online October 25, 2023. doi:10.1001/jamanetworkopen.2023.39723

Data
Data available: No

Additional Information
Explanation for why data not available: Data will not be made publicly available, but data are available to qualified researchers through the Kaiser Permanente (KP) Research Bank, contingent upon project approval by the KP Research Bank Access Review Committee, IRB approval, and execution of a Materials and Data Transfer Agreement.
